# Artificial Intelligence for the Future Radiology Diagnostic Service

**DOI:** 10.3389/fmolb.2020.614258

**Published:** 2021-01-28

**Authors:** Seong K. Mun, Kenneth H. Wong, Shih-Chung B. Lo, Yanni Li, Shijir Bayarsaikhan

**Affiliations:** Arlington Innovation Center:Health Research, Virginia Tech-Washington DC Area, Arlington, VA, United States

**Keywords:** artificial intelligence, radiology, CNN, productivity, integrated diagnostics, workflow

## Abstract

Radiology historically has been a leader of digital transformation in healthcare. The introduction of digital imaging systems, picture archiving and communication systems (PACS), and teleradiology transformed radiology services over the past 30 years. Radiology is again at the crossroad for the next generation of transformation, possibly evolving as a one-stop integrated diagnostic service. Artificial intelligence and machine learning promise to offer radiology new powerful new digital tools to facilitate the next transformation. The radiology community has been developing computer-aided diagnosis (CAD) tools based on machine learning (ML) over the past 20 years. Among various AI techniques, deep-learning convolutional neural networks (CNN) and its variants have been widely used in medical image pattern recognition. Since the 1990s, many CAD tools and products have been developed. However, clinical adoption has been slow due to a lack of substantial clinical advantages, difficulties integrating into existing workflow, and uncertain business models. This paper proposes three pathways for AI's role in radiology beyond current CNN based capabilities 1) improve the performance of CAD, 2) improve the productivity of radiology service by AI-assisted workflow, and 3) develop radiomics that integrate the data from radiology, pathology, and genomics to facilitate the emergence of a new integrated diagnostic service.

## Introduction

Radiology was one of the first specialty in healthcare to adopt digital technology. Since the 1970s, radiology has adopted many new digital imaging modalities such as Computed Tomography (CT), Magnetic Resonance Imaging (MRI), Positron Emission Tomography (PET), Computed Radiography (CR), Single Photon Emission Computed Tomography (SPECT), Digital Ultrasound, Digital Mammography and many others. These digital images were initially printed on films for interpretation, sharing, and archiving. As digital technologies for data capture, data storage, image display, and transmission improved, radiology operations began to convert to a filmless digital environment in the late '90s (Mun et al., 1993; Mun et al., 2007). Today, x-ray films are gone, and the PACS manages all radiological images (Alhajeri et al., 2017). This massive investment in digital technology transformed the radiology service and made radiology images ubiquitous throughout all aspects of healthcare (Hricak, 2018). Digital radiological images enabled the development of many new image-guided surgeries and radiation oncology. Radiology became global as teleradiology was the first successful telemedicine application globally (Mun et al., 1998; Mun and Turner, 1999). Teleradiology, often globally, is a significant portion of radiology operations in the US. Radiology services accumulate massive digital images in their archives, some in the cloud, that laid a technological and human infrastructure for the next digital transformation based on machine learning (ML) and artificial intelligence (AI).

During the mid-'80s, the radiology community began to explore computer aided diagnosis (CAD) as a possible to aid radiologists (Doi, 2007). Since the mid-2010s, there has an overwhelming interest in machine learning techniques in almost all fields involving data classification or analysis. The number of publications using ML has been exponentially increasing from a few thousand per year in the early 2000s to about 35,000 per year in 2018; and nearly 85% were in neural networks, based on Scopus data (Perrault et al., 2019). Several versions of the neural network technique also were used for drug discovery, computational biology, quantum chemistry, autonomous cars, geology, astronomy, and many others.

Many CAD tools were developed in radiology community, with good performance in terms of sensitivity and specificity. However, most of them remained in research labs, and they did not become an integral part of the radiology service. An earlier success in the use of CAD in digital mammography for breast cancer screening generated much excitement in the community for wider clinical adoption of CAD tools. Some speculated that these intelligent systems would soon replace radiologists.

The National Science and Technology Council of the US published a research and development roadmap for medical imaging (Interagency Working Group on Medical Imaging, Committee on Science, and National Science and Technology Council, 2017). The report envisions changes in medical imaging in 4 general areas; 1) patient referral to imaging service, 2) development and use of high-value imaging capability, 3) use of advanced computation and machine learning, and 4) promoting best practice in medical imaging including reorganizing workflows to improve productivity.

In the article, we discuss AI research in medical imaging from a clinical adoption perspective for patient care and suggest several pathways through which AI will be demanded by radiology as it undergoes the next generation digital transformation toward integrated diagnostic service.

### Operational Description of Radiology Service

Radiology service is a very complex operation that includes many inefficiencies. There is a great need to improve overall workflow and productivity. The radiology department provides clinical services to referring physicians and patients by managing a complex workflow involving many layers of people, various technology, many types of time-sensitive information. On any given day, a typical radiology department will conduct more than 50 different types of imaging studies covering all body parts using dozens of imaging modalities, including CT, MRI, ultrasound, nuclear medicine, positron emission tomography, and various conventional radiography systems. The acquired images are managed by a picture archiving and communication system (PACS) and radiology information system (RIS) (Boochever, 2004). Orders for imaging studies from the referring physicians are placed by referring physicians based on the patient's medical history and symptoms. A radiologist determines imaging protocol suitable to address the clinical question. When an imaging study is completed at the imaging system, the PACS will collect all images and generate a worklist for each radiologist based on departmental policies/procedures and the radiologist's specialization (Hricak, 2018).

The radiologist's work has three parts: interpreting and analyzing images, generating reports, and providing further consultation for referring physicians and patients (Halsted and Froehle, 2008). In academic departments, training residents and fellows is also a significant responsibility. The interpretation (reading) time varies greatly depending on the types of study. The radiologists are highly skilled and very fast at detecting abnormalities in the image (Forsberg et al., 2017). The reading times in radiology has been steadily increasing. The imaging devices have improved, and they generate increasingly more images per study. For example, the average number of images for a CT exam increased from 82 images in 1999 to 679 images in 2010. For MRI, the numbers increased from 164 images to 570 images, respectively (McDonald et al., 2015). The image interpretation often requires examining previous studies and comparing them to the current study to determine if the patient has gotten better or worse. These comparison analyses are carried out manually by a radiologist, which can be very time-consuming (Schemmel et al., 2016; Doshi et al., 2018).

Once the radiologist has completed their interpretation of the study, the results are generally recorded on a voice recognition system (a component of the PACS/RIS) to generate written reports that become part of the patient record (Boochever, 2004). In some cases, the report is sufficient by itself. In other cases, the report becomes part of a more complex analysis (Kahn et al., 2009). For example, a patient might have multiple radiology exams, blood chemistry analysis, and tissue sampling, all of which need to be combined to create a comprehensive diagnosis.

Most of the previous efforts to apply AI to radiology in terms of CAD so far have focused exclusively on the interpretation of single images or a single series of images. In comparison, this is an important task, only a fraction of how a typical radiologist spends their time. When the image's reading/analysis can become a part of subsequent decision-making, radiologists would participate in the decision making beyond the reports (Brady, 2017). In essence, AI in radiology has historically tackled challenging yet narrow problems (Schemmel et al., 2016; Hosny et al., 2018). We argue that a refocusing of AI onto different aspects of the radiology workflow and medical error reduction will generated more demand and adoption by radiology community. (Dikici et al., 2020; Montagnon et al., 2020).

### Clinical Adoption Case Study: Breast Cancer Screening and Diabetic Retinopathy

One has to distinguish between CAD, CADx, and CADe, a family of AI tools based on convolution neural network (CNN). The concept of computed diagnosis (CAD) research in medical imaging has evolved into two distinct clinical applications; computer aided diagnosis (CADx) and computer aided detection (CADe). CADx means the computer provides a diagnosis for physician review. On the other hand, CADe means the computer highlights the area of concern (i.e., cancer) for further diagnostic evaluation without providing a diagnosis. The CADe is used to screen cancers such as lung cancer or breast cancer for the asymptomatic but higher risk population. If cancer is suspected from the screening study, the patients will undergo a higher precision diagnostic study. The CADe is a much more challenging problem compared with CADx.

We will review the clinical adoption experience of two different use of AI, breast cancer screening and diabetic retinopathy screening to highlight that clinical adoption is a multifaceted issue beyond technical success in laboratories.

### CADe in Screening Mammography for Cancer Detection

Screening mammography is an ideal application for CADe because it has to review many cases by a limited number of radiologists trained in mammography. It is a single type of exam, and the most basic output is a simple yes (disease present) or no (disease absent). FDA approved the first CADe for mammography in 1998, but its adoption rate was initially less than 5% (Lehman et al., 2015). However, as breast cancer screening became more popular as part of a government policy to promote women's health, there was a shortage of skilled radiologists for mammography. To address the shortage, which resulted from low reimbursement rates for reading mammograms, the Center for Medicare and Medicaid Services (CMS) allowed higher reimbursement rates for using CADe in screening mammography (Gold et al., 2012). This financial incentive has dramatically increased the adoption of CADe for mammography. In fact, in the US today, most breast cancer screening mammograms are interpreted by radiologists with CADe assistance. It is highly unlikely that additional reimbursements would be allowed to use AI tools in radiology in the future.

The widespread use of mammography CADe and the large number of exams performed each year allowed assessment of CADe's impact on mammographic interpretations' accuracy. One of the largest, a study involving 271 radiologists and 323,973 women between Jan 2003 and Dec 2009, compared reading mammograms with and without CADe. The study concluded that CADe does not improve mammography's diagnostic accuracy (Lehman et al., 2015).

A more recent study by Schaffter and colleagues conducted a crowd-sourced trial on the use of deep learning in digital mammography involving 300,000 mammograms from the US and Sweden. The project had 126 teams from 44 countries to see if they could meet or beat the radiologist’s performances. They concluded that AI tools again did not perform better than radiologists (Schaffter et al., 2020).

### CADe Use to Screen Diabetic Retinopathy to Prevent Blindness

Diabetic retinopathy is an eye disease when high blood sugar levels of a diabetic patient can cause damage to blood vessels in the retina. Undetected and untreated the patient can become blind. Screening for diabetic retinopathy is an effective way to prevent the blindness. A special camera takes images of the blood vessels in retina. Interpretation of these images requires special expertise, thus it has been a interests of AI community to develop CAD systems.

Recently Google Corporation deployed its AI tool to detect diabetic retinopathy in Thailand. Initial development and testing involved 3,049 patients. In 2018, they deployed the system at 11 clinics in Thailand, involving 7,600 patients (Beede et al., 2020). This large-scale prospective study was halted mainly because of persistent image quality problems. The system performed poorly, mainly due to the variability of retinal scan images obtained at different nurses' different settings (Beede et al., 2020). The success in the lab did not translate well in real-life situations. Initial deployment of an AI system for lung cancer screening experienced a similar situation in dealing with a considerable variability of image quality of real-life clinical cases (Worrell, 2020). Pre-processing of image normalizing before AI application may be an essential step for scalable clinical deployment.

Currently, 46 AI algorithms have approvals from Food and Drug Administrations and Conformité Européenne (CE) (Tadavarthi et al., 2020). The approval process consists of clinical trial demonstration clinical safety and efficacy, often involving receiver operating curve analysis of sensitivity and specificity. The approval, however, does not guarantee successful clinical adoption. Global adoption of these tools is still a few years away. Since there are no insurance reimbursements, except digital mammography in the US, for the use of AI software, return on investment must be assessed based on significant quality improvement or efficiency improvements. AI tools have not shown significant improvements in the quality of diagnosis and operational efficiency for cost savings. Such improvement may require significant changes in the radiology department functions and possible re-configurations of PACS often owned by vendors other than current AI vendors.

### The Technology of CNN and Computer-Aided Diagnosis

Artificial intelligence (AI) is the capability of the machine to imitate intelligent human behavior. In contrast, machine learning (ML) is a subfield of AI that allows the machine to learn from data without being explicitly programmed (Soffer et al., 2019). The concept of neural networks emerged from the biologic neuron system. A neural network in the visual cortex can detect the edges of an object seen by the retina. When the receptors' inner parts are activated simultaneously, the cell neutron integrates the signals and transmits an edge detection signal. An artificial neural network (ANN) is composed of interconnected artificial neurons. Each artificial neuron implements a simple classifier model, which outputs a decision signal based on a weighted sum of evidence, and an activation function integrates signals from the neurons. An ANN system can be built with thousands of these basic computing units. The system can be trained by computing these weights using a learning algorithm where pairs of input signals and desired output decisions are presented, mimicking brain functions. An individual artificial neuron is a simple neural network; however, multilayer perceptron can model complex nonlinear functions. The deep learning (DL) concept is based on the use of multilayer architecture of multilayer perceptron. In medical imaging, the number of layers tends to be in the range of dozen.

The convolution neural network (CNN) consists of a series of convolution layers equivalent to compositional convolution layers with a set of large kernels. In effect, a CNN acts as a feature learning based on spatial features with multiple channels (Lo et al., 1995; Lo et al., 2018b; Lo et al., 2018c). However, common difficulties in traditional CNN approaches for medical imaging can be grouped into three categories; 1) inability to separate normal from ill-defined abnormal structures, 2) inability to differentiate disease patterns, particularly in subtle cases from a broad spectrum of normal structures, and 3) inability to establish an integrated system between compositional and divide-and-conquer models.

### Limitation of Current Generic CNN

Many ML open-source packages such as Tensorflow, Keras, Caffe, and others featuring CNN have been widely used. The core algorithms of CNN in all these packages were designed for general image pattern recognition. They were initially developed for the recognition of alphanumerical handwriting. General image pattern recognition relied on essential graphic pattern features (e.g., edges) and orientation-dependent but size-independent in many situations. On the other hand, medical image pattern recognition should rely more on gray intensity distribution and is orientation independent but size-dependent. Also, some users have experienced inconsistent results from the current CNNs and have tried to use many versions converted from the same input as a part of the augmentation strategy to increase the training samples and stability (Lo et al., 1998).

### Improving CNN for Medical Imaging

An ordinary CNN using unconstrained kernel weights entirely based on the backpropagation training (Lo et al., 2018d). The use of rotational and translational versions of each input vector as data augmentation was developed by the authors (Lo et al., 1993). However, many investigators reported that the current method requires a long training time and produces unstable results (Lo et al., 2018b; Lo et al., 2018c).

The current CNN software should be redesigned for medical imaging pattern recognition by (i) the use of an activation function without suppression of the composed signal and (ii) the use of symmetric kernels. This is because current activation functions (Relu, Leaky Relu, sigmoid, and Tanh) used in general CNN tools are signal suppression functions (i.e., df/dx < 1). When using them multiple times through multiple convolutional layers, only edge patterns with very few gray value features remained in the feature maps at the end of convolution/activation processing for final classification. These are not acceptable intermediate outcomes for many medical images where subtle gray value differences are used for discerning possible disease characteristics (Lo et al., 1995; Lo et al., 1998).

The symmetric kernels within CNN should be used to stabilize the CNN output consistency. The use of kernels with dihedral symmetry of order 8 (Dih4) is an example with a minimum number of free parameters as element coefficients are symmetric with respect to each corner wedges. In other words, elements on other wedges corresponding to the Dih4 symmetric element positions in the wedge would share the same value. The use of symmetric kernels can be expanded to wavelet decomposition. Though it is different from an ordinary convolution process, each compartment's biorthogonal kernels may be different. However, t the absolute value in each element of the kernel is the same. The multi-dimensional wavelet decomposition is made by a one-dimensional convolution process and down sampling one half at a time. The total number of free parameters is much less (the number of elements in 1D kernel plus 1, divided by 2). In effect, kernels to produce low-low (LL) and high-high (HH) compartments are Dih4 transformation-identical (TI) kernels. Kernels to produce low-high (LH) and high-low (HL) compartments are Dih4 TI with an odd number of elements but are anti-symmetric (i.e., 180^o^ rotation TI) with an even number of elements. Since each compartment is processed through an independent pipeline in the neural network process, for the latter situation, there is still room to make signals from LH and HL be Dih4 TI, if desired. This can be done by inserting a reflective symmetry kernel in each of these two compartment pipelines (Lo et al., 2018d).

With symmetric kernels, such as the dihedral symmetry of order 8, the intermediate results throughout all convolutional layers would be equivariant for original input and 90^o^ rotation increment as the flipping version. With this equivariant property on all convolutional layers, the CNN would produce identical output for all eight input image versions. In summary, the CNN can be treated as a whole function of an input vector Vi (i.e., a 2D image or 3D volume), and the output vector can be expressed as Vo = CNN(T[Vi]) = CNN(Vi) as long as T[K] = K within each of the CNN convolutional processes, where Vi and Vo are input and output vectors, respectively. K denotes the convolution kernel and T[.] is a transformation function. This equivariant property at all convolutional layers can be extended for the CNN to produce identical output results for any arbitrarily rotated images by merging the convolution processing before the classification section in the CNN. The use of symmetric kernels in the convolutional layers in the CNN would be a more appropriate tool to systematically produce highly stable results (Lo et al., 2018d).

### Open Source CNN

There have been increasing concerns about the ethics, ability to explain and transparency of AI technology (Tang et al., 2018; ESR, 2019), especially in healthcare. These concerns are partly due to difficulties in understanding underlying theories, methods, and assumptions used to generate systematic bias results. In this scenario, the use of open-source software (OSS) strategy could help address some of these concerns because, by definition, OSS offers greater transparency of the technology and opportunities for community-based collaboration.

The OSS concept started in the 1980s as a social movement and a philosophy for software development and distribution (Levine and Prietula, 2014). OSS is defined as a software code made available under a legal license in which the copyright holder provides (depending upon the specific terms) various rights to the licensees to study, change, improve and re-distribute the code without any fees. Today there are many different types of OSS licenses depending on the copyright holders' interests and intentions (Fosfuri et al., 2008; Opensource.org, 2020). These licenses range from permissive licenses such as Apache-2.0 to strongly protective licenses such as general public license (GPL). OSS is typically available as-is; however, it can be made into commercial products with additional services such as warranty, training, documentation, and maintenance under various commercial contracts.

Some of the more popular packages include TensorFlow, Keras, PyTorch, Caffe2, and many others. They all have varying strengths and weaknesses, depending on users' needs. Keras and TensorFlow have a common or similar core, but Keras is much easier to use with limited options than TensorFlow. PyTorch is fast and flexible for experimentation, and it is tightly integrated with the Python language. An extensive table of available software with detail comparison can be found at Wikipedia (Multiple-Editor, 2020).

These OSS packages are developed and sponsored by various organizations and individuals for their use cases and applications other than medical imaging. However, the packages are initial starting platforms for imaging research. These open source packages should be optimized to be suitable for meaningful medical imaging research as discussed in the earlier section. Additionally, these open source codes' users should form or join collaborative communities based on shared medical imaging interests.

### Application of CNN in Medical Imaging:

For research in supervised learning such as CNN, the success depends on three technical factors: 1) underlying science and technology of the code, 2) learning supervised by subject matter experts, and 3) the quantity/quality of data.

Another crucially important factor in CNN research is imaging expertise, both clinical and physic of imaging (Giger et al., 2008). Unlike common everyday objects in AI research, the research team in medical imaging AI has to understand the clinical significance of images and imaging physic.

### Availability of Data and Realistic Mix of Data

There are two data issues: access to a sufficient volume of data and enough data diversity representing a realistic case mix of the clinical operational environment (Yamashita et al., 2018).

The imaging data requirement in radiology is relatively modest, less than 10,000 cases per disease category. In the case of the recent AI tool development for lung cancer screening with CT images, approximately 2,000 cases consisting of 300,000 CT images were sufficient for training, and approximately 300 cases of 45,000 images with about 20% subtle cases tested by more than 10 radiologists were sufficient for an FDA specified clinical trial (Lo et al., 2018a). For different disease types and imaging modalities, these numbers would be different. If the clinical problem to be addressed has many subtle features, the data volume required would be much higher.

For an AI algorithm to be clinically useful, it must be trained on data that appropriately represent the patient population's variance and diseases' presentation. In a routine data collection effort, the majority of available cases show disease patterns, which are considered relatively easy cases. The cases of subtle disease patterns are relatively rare and thus challenging to collect. It is essential to have a mix of subtle cases in the image archive. If one has a disproportionately large number of similar or easy cases, the system will show bias (Lo et al., 1995; Hosny et al., 2018). In supervised learning, algorithms such as CNN learn from labeled data. When the number of categories and/or patterns to be differentiated increases, the required data volumes would increase. The problem of having more dimensions, yet small data volume, can result in overfitting contributing to low generalizability and scalability (Yamashita et al., 2018; Mutasa et al., 2020).

### Quality of Data: Image Quality

The performance of current CNN is fragile, dealing with varying image quality. In fact, in any data science project, one can spend a significant amount of effort to "clean" the data. The same is true in imaging. The data must be of sufficient quality and acquired with uniform parameters to make certain that conclusions can be validated. The image quality can vary depending on the time and day of imaging, image protocol, imaging system set up, patient conditions, and clinical practice standards in different departments (Worrell, 2020). While human vision is good at reading through the images of varying qualities, AI tools are generally not (Tang et al., 2018). One important task to produce a systematic image AI performance is image pre-processing, including optimization of image quality, noise reduction, clutter removal, and enhancement of essential features for differentiation. Various AI tools are used to standardize image quality (Zhu et al., 2017; Mazurowski et al., 2019).

A radiology AI tool for screening or diagnosis of a disease is usually comprised of several components: 1) pre-processing such as image normalization, 2) image segmentation or region of interest (ROI) extraction, and 3) potential disease pattern identification and classification. Various algorithms have been applied to each of these AI sub-components. However, there is a trend to use a fully CNN-based algorithm such as U-net for image segmentation and use a classification CNN- based algorithm for identification and classification of the disease aiming at the ROI. Alternatively, radiomics based classification can be employed on the ROI.

### Data Labeling

In radiological imaging, the supervised learning approach is the most popular tool, and it requires labeled data for training and validation. The labeling of images must be done manually by expert radiologists. This process is very labor-intensive and very costly. The truth panel for images is established by having 2 out of 3 radiologists agreeing on the diagnoses and clinical determinations (Lo et al., 2018a).

### Research Environment - Access to Research Resources

The role of AI will be different in different parts of the world. The AI tools developed for one region of the world using data from that region may not be useful in other regions with different disease prevalence, limited infrastructure, and different healthcare systems.

Collection and curation of images and related data could face several obstacles such as management of privacy, confidentiality, and the question of ownership. In recent years, the realization of clinical images' possible commercial values makes access more costly and difficult. When images need to be collected from multiple organizations, the data sharing process can become more complicated. International collaboration can be difficult when certain countries do not allow clinical data movement beyond their national borders (Prior et al., 2020). One technical solution for such a situation can be a federated learning system where data remains in place while processing code and processed results can move around (Konečný et al., 2016)

Government agencies and various consortia have established a growing number of open access data repositories to facilitate better access to clinical image data for research. One of the best-known such repositories is the Cancer Imaging Archive (TCIA) (Prior et al., 2013; The Cancer Imaging Archive (TCIA), 2020). It is a publicly available information repository for data about cancer, mostly radiology and pathology data acquired by the lung cancer screening project involving 26,722 participants from 2002 through 2004. It contains 22.3 TB of data. The types and volume of data in the archive are increasing rapidly.

### Research and Development Environment in Resource-Limited Regions

The research and development and eventual adoption of AI for medical decision making in global health and low-resource settings are hampered by insufficient infrastructure (Mollura et al., 2020).However, it is essential that local radiology and clinical community, resource-poor or not, have to develop and validate AI tools suitable for their environment. In the resource-poor regions with limited infrastructure, technical and human, such participation could be difficult. However, the research communities in the world's resource-limited regions can access many global imaging AI research resources. The Radiological Society of North America website has a vast amount of information. Many of the AI software is freely available as open-source at no cost to the users. The cancer imaging archive (TCIA) of the National Cancer Institute of the US has many curated radiological images to support imaging research. Most of the CADe products for lung cancer screen started using the openly available images in TCIA, which holds the CT images from the national lung cancer screening trial.

### Future of Radiology Service and Radiomics

The digital transformation of radiology services will continue and accelerate. Analog film is gone, and modern imaging systems have evolved far beyond the slow and primitive early MRI and CT systems. PET was only a research tool at a few centers but is now becoming available at small community hospitals. Hybrid scanners that combine multiple modalities and can operate in different healthcare settings will become readily available. The whole-body scanner that could do MRI or CT or PET will be available where the patients receive care (Pichler et al., 2008; Nensa et al., 2018). PACS, teleradiology, and Radiology Information Systems all changed the radiology practice.

Nevertheless, the radiology department structure has not fundamentally changed in 30 years (Kim and Mansfield, 2014). The radiology department continues to operate as a centralized resource to which patients come to complete the study, and radiologists dictate and distribute the reports (Ondategui-Parra et al., 2004). This operational model may soon see some changes.

For the next 30 years of radiology in the future, the pace of change will accelerate. The cost of computing will continue to decrease, and connectivity will be fast and ubiquitous. Radiology and the field of diagnosis will evolve together with pathology. Diagnostic imaging, clinical pathology, and genomics could merge as an integrated diagnostic service that can integrate the various reports from these subspecialty sections and synthesize a coherent diagnosis that is communicated to the appropriate physicians with more actionable specifics. Such an integrated system will allow a rapid on-site point of care diagnosis, rather than the serial process of today involving multiple appointments over many days and weeks.

Some expect that a new profession of clinical diagnosticians, who integrate the work of radiologists and pathologists with other specialists with increasing reliance on AI assistance, will begin to grow (Lundström et al., 2017). Pathologists are already doing biopsies under image guidance. There are movements toward the integration of professional service. Simultaneously, there are parallel significant scientific and technical developments underway in radiomics and pathomics that can facilitate this historical evolution.

Radiomics and pathomics are part of quantitative imaging that attempts to extract additional information from radiology and pathology images that may not be visible via visual inspection (Saltz et al., 2017). Radiomics attempts to extract features from radiological images that quantify its phenotype characteristics in an automated high-throughput manner (Fan et al., 2020). Pathomics attempts to extract similar information from pathology images. These two approaches will meet at a shared space to support personalized medicine. It has been hypothesized that such analysis may help prognosticate, predict treatment outcomes, and assess cancer tissue malignancy.

The value of AI in radiomics is two-fold. First, AI can be used for automated image analysis at scale, enabling rapid evaluation of hypothetical radiomic features. Whereas comparison studies involving human radiologists should take into account for a wide range of ergonomic and perception factors (such as the required number of readers or the need to provide time between different readings of the same image), a comparison of radiomics algorithms is only limited by computational speed and power. Similarly, new features can easily be tested against existing data sets. Secondly, unsupervised learning methods can be used to search for new radiomic features that might be very different from what would be noticed by a human observer. The AI can take on the discovery and find new and useful patterns within the existing imaging data (Miles, 2020; Prior et al., 2020).

One fundamental issue in radiomics has to address is the standardization of the factors and processing involved in the quantitative analysis. The Image Biomarker Standardization Initiative (IBSI) is a new organization to address many challenges in 4 different specific areas, 1) standard nomenclature and common radiomic features, 2) radiomics image processing schemes, 3) provide data sets for validation and calibration, and 4) set of reporting guidelines (Zwanenburg et al., 2020). This group defined 174 radiomics features commonly used to quantify the morphologic characteristics and numerous others needed to define the quantitative information. The group tries to standardize the image processing steps of data conversion, post-acquisition processing, segmentation, interpolation, masking, and others (Zwanenburg et al., 2020)(Zwanenburg et al., 2020). Such standardization is expected to make radiomics and pathomics clinically useful and scalable for the integrated diagnosis service (Kuhl and Truhn, 2020).

## Conclusion

The clinical adoption of AI can be driven by either technology push or market pull (Chidamber and Kon, 1994; Di Stefano et al., 2012). Technology push arises when a new idea or new tool creates a capability that did not previously exist. The market pull is defined by the need to address pain points, inefficiencies, and problems with the current way of doing business. Ideally, these two forces synergistically combine to accelerate technology development and deployment. Over the last 30 years, radiology has benefited from this combination to develop teleradiology, PACS/RIS, and advanced imaging modalities. However, much of CAD's early development has been a technology push; it was usually not well aligned with clinical needs. We have proposed that a better alignment could arise from focusing on the radiology workflow – which includes many tasks beyond image interpretation – and the need to create an integrated diagnostics service combining radiological images, pathological data, and genomics (Santos et al., 2019).

**FIGURE 1:**
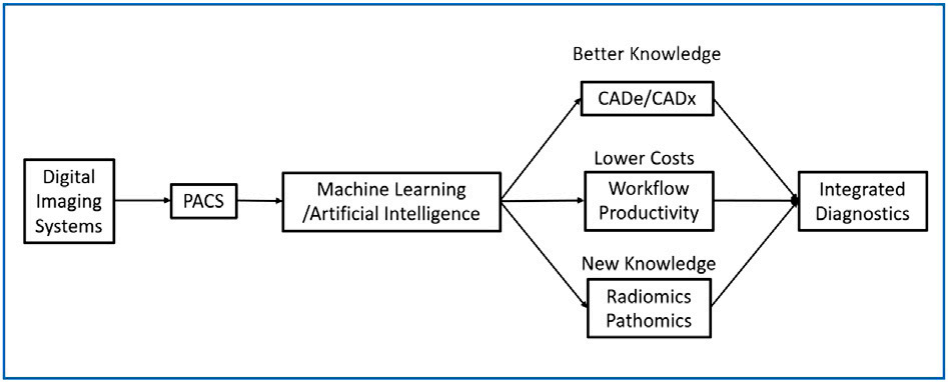
Digital Transformation of Radiology with 3 Categories of Application of ML/AI.

We envision three AI trajectories in radiology, as shown in Figure 1. First, AI will undergo advances in CADe and CADx to make image interpretation better and faster. Despite the significant progress in developing CNN algorithms, there are still many areas for improvement as proposed in this paper. Second, a variety of AI tools, supervised learning and unsupervised learning, will be needed to improve workflow and increase productivity, and, at the same time, reduce the cost of operation. This operationally focused research will require a holistic understanding of radiology operations. Third, quantitative imaging, including radiomics, pathomics, and genomics, will emerge and become a standardized approach for integrated diagnostics. In summary, we predict that AIs will facilitate the merging of disparate medical and scientific domains into an integrated diagnostic service for personalized precision medicine.

## Author Contributions

SM designed the document and integrated different ideas into a single document. KW drafted the section dealing with the clinical adoption of AI and radiomics and edited the final document. SL drafted the section on CNN and data needs. YL did a literature review and developed the list of relevant references. SB was responsible for developing radiology workflow issues.

## Conflict of Interest

The authors declare that the research was conducted in the absence of any commercial or financial relationships that could be construed as a potential conflict of interest.
